# A Case of Cyanamide-Ethanol Reaction Leading to Atrial Fibrillation and Heart Failure

**DOI:** 10.7759/cureus.86699

**Published:** 2025-06-24

**Authors:** Toshimitsu Kobori, Ginga Suzuki, Saki Yamamoto, Yui Shimanuki, Kohei Ishikawa

**Affiliations:** 1 Critical Care Center, Toho University Omori Medical Center, Tokyo, JPN

**Keywords:** acetaldehyde toxicity, alcohol dependence, congestive heart failure due to atrial fibrillation, cyanamide, cyanamide-ethanol reaction

## Abstract

The cyanamide-ethanol reaction (CER) is a rare but potentially life-threatening adverse event caused by the inhibition of aldehyde dehydrogenase (ADLH), leading to the accumulation of acetaldehyde after alcohol intake. This reaction is characterized by facial flushing, hypotension, tachycardia, nausea, and vomiting. We report the case of a 65-year-old male who developed new-onset atrial fibrillation (AF) and congestive heart failure (CHF) following moderate alcohol consumption while on cyanamide therapy for alcohol dependence. About twelve hours after taking cyanamide, he consumed alcohol equivalent to 32 grams of ethanol and soon experienced tachycardia, dyspnea, and nausea. Electrocardiography revealed AF, and subsequent imaging and laboratory tests confirmed the diagnosis of CHF. While vasodilatory shock related to CER has previously been documented, to our knowledge, this is the first reported case of CER-induced AF and CHF. This case underscores the necessity of including CER as a differential diagnosis in patients presenting with acute circulatory or respiratory failure while undergoing cyanamide therapy.

## Introduction

Cyanamide is a widely used alcohol deterrent agent in the treatment of alcohol dependence, particularly in Japan, where it remains one of the primary pharmacological options. Unlike disulfiram, which has largely fallen out of favor due to its adverse effect profile and limited availability in some regions, cyanamide is still commonly prescribed in Japan owing to its oral liquid formulation, tolerability, and long-standing clinical familiarity. It is often administered in supervised settings or as part of structured relapse prevention programs in both inpatient and outpatient contexts.

Cyanamide acts as a reversible inhibitor of aldehyde dehydrogenase (ALDH), an enzyme responsible for metabolizing acetaldehyde, a toxic intermediate produced during ethanol oxidation. When ALDH is inhibited, acetaldehyde accumulates in the bloodstream, leading to a constellation of unpleasant symptoms--facial flushing, nausea, vomiting, tachycardia, and hypotension--collectively known as the cyanamide-ethanol reaction (CER) [1-3].

Although the pharmacodynamics of cyanamide are well documented, the clinical severity of CER varies widely. Most cases are mild to moderate, but some can progress to serious complications, such as lactic acidosis or vasodilatory shock, and may even be fatal [3-5]. Despite extensive documentation of CER's typical manifestations, no previous reports have described atrial fibrillation (AF) or congestive heart failure (CHF) as consequences of this reaction.

Additionally, it is known that acute alcohol intake, even in the absence of deterrent agents, can trigger transient cardiac arrhythmias, a phenomenon termed “holiday heart syndrome.” This syndrome most commonly manifests as AF in individuals without underlying cardiac disease. However, the interplay between cyanamide use and alcohol-triggered AF has not been previously explored.

We herein describe a rare case of a patient who developed both AF and CHF following the consumption of a moderate amount of alcohol 12 hours after cyanamide administration. This case highlights the potential for severe and previously unreported cardiac complications associated with CER and underscores the importance of considering such risks in clinical practice.

## Case presentation

A 65-year-old male with no prior cardiovascular history presented to the emergency department with tachycardia and hypoxemia following alcohol ingestion. He had commenced treatment with oral cyanamide (100 mg/day) 10 days prior to the incident for alcohol relapse prevention. On the day of presentation, he took cyanamide at 6:00 AM as prescribed and subsequently ingested 200 mL of 20% shochu (approximately 32 grams of ethanol) at around 6:00 PM. Within one hour, he developed palpitations, shortness of breath, and nausea, and emergency services were contacted.

Upon arrival by emergency medical services, he exhibited a respiratory rate of 42 breaths per minute and a heart rate of 150 beats per minute. At presentation to the emergency department, his vital signs were as follows: Glasgow Coma Scale score of 15 (E4V5M6), blood pressure 77/59 mmHg, heart rate 155 bpm, respiratory rate 38/min, and SpO₂ of 99% under 6 L/min of oxygen supplementation.

**Figure 1:**
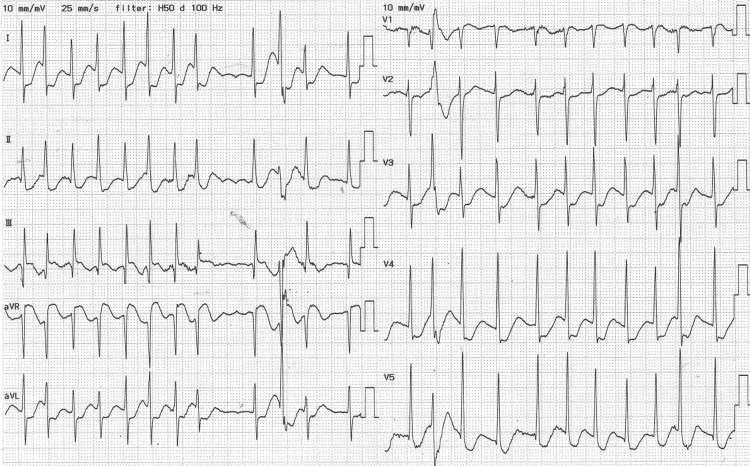
Electrocardiogram on arrival. The 12-lead ECG obtained at presentation shows atrial fibrillation with a rapid ventricular response (heart rate approximately 153 bpm). No distinct P waves are observed, and the R-R intervals are irregular, consistent with a diagnosis of tachyarrhythmic atrial fibrillation.

Laboratory evaluation revealed respiratory compensation for lactic acidosis and significant hypokalemia (serum potassium 2.6 mmol/L). B-type natriuretic peptide (BNP) was markedly elevated at 1603.3 pg/mL (Table 1). A 12-lead electrocardiogram demonstrated AF with a heart rate of 153 bpm (Figure 1).

**Table 1:** Laboratory findings on admission to our hospital. *Abnormal. WBC: white blood cell count, CRP: C-reactive protein, Hb: hemoglobin, Plt: platelets, AST: aspartate aminotransferase, ALT: alanine aminotransferase, ALP: alkaline phosphatase, γ-GTP: gamma-glutamyl transpeptidase, T-Bil: total bilirubin, Alb: albumin, BUN: blood urea nitrogen, Cre: creatinine, Na: sodium, K: potassium, Cl: chloride, PT: prothrombin time, APTT: activated partial thromboplastin time, BNP: B-type natriuretic peptide.

Laboratory examination	Result	Reference range
WBC	14,400/μL*	3500-9000/μL
Hb	12.7 g/dL*	13.5-17.5 g/dL
Plt	320,000/μL	150,000-350,000/μL
CRP	5.3 mg/dL*	0.0-0.3 mg/dL
AST	43 U/L*	10-40 U/L
ALT	26 U/L	5-45 U/L
ALP	65 U/L	100-350 U/L
γ-GTP	323 U/L*	10-70 U/L
T-Bil	0.8 mg/dL	0.2-1.2 mg/dL
Alb	3.7 g/dL*	3.8-5.3 g/dL
BUN	6 mg/dL*	8-20 mg/dL
Cre	0.91 mg/dL	0.7-1.1 mg/dL
Na	139 mEq/L	135-145 mEq/L
K	2.6 mEq/L*	3.5-5.0 mEq/L
Cl	102 mEq/L	98-108 mEq/L
Calcium (corrected)	9.5 mg/dL	8.5-10.5 mg/dL
Serum amylase	105 U/L	40-120 U/L
Glucose	177 mg/dL*	70-109 mg/dL
PT	9.4 seconds*	10-13 seconds
APTT	25.4 seconds	25-35 seconds
BNP	1603.3 pg/mL*	0-18.4 pg/mL
Venous blood gas values		
pH	7.46	7.35-7.45
PaCO_2_	25.8 mmHg*	35-45 mmHg
PaO_2_	42.3 mmHg*	30-40 mmHg
HCO3-	17.8 mmol/L*	22-26 mmol/L
Standard base excess	-5.4	-2 to +2
Lactate	6.4 mmol/L*	0.5-2.2 mmol/L

**Figure 2:**
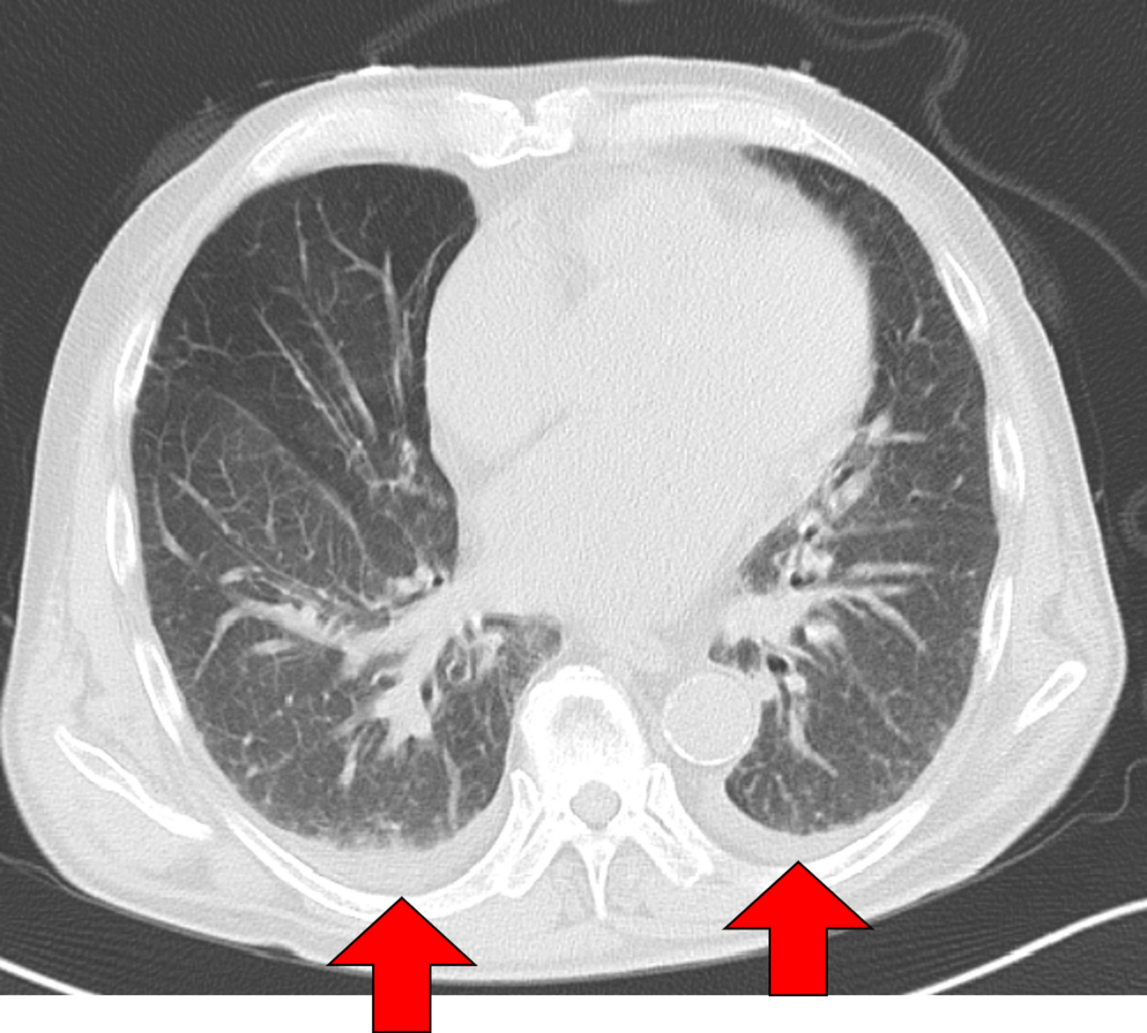
Chest CT on admission to our hospital showing pleural effusion (red arrow).

Initial resuscitation with a rapid infusion of 30 mL/kg of intravenous fluids did not yield hemodynamic improvement. Continuous infusions of landiolol and norepinephrine were initiated, resulting in conversion to sinus rhythm and stabilization of blood pressure. Due to persistent tachycardia, echocardiographic assessment was inconclusive. Chest computed tomography (CT) revealed bilateral pleural effusions (Figure 2), consistent with a diagnosis of CHF.

**Figure 3:**
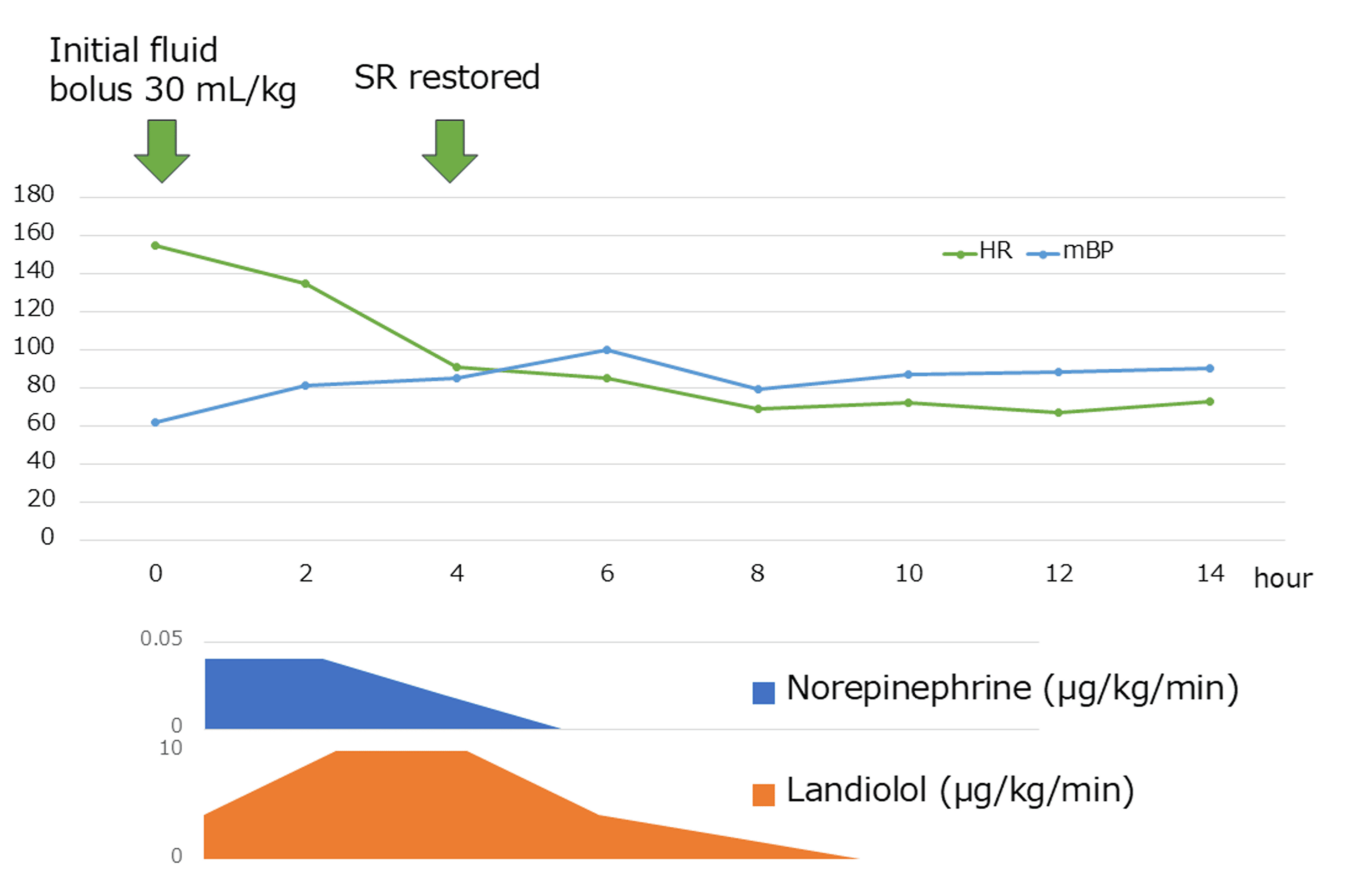
Hemodynamic response and medication timeline within the first 14 hours of hospitalization. The upper panel shows the patient's heart rate (HR) and mean blood pressure (mBP) trends during the first 14 hours after admission. The lower panel depicts the infusion rates of norepinephrine and landiolol. At hour zero, an initial rapid infusion of 30 mL/kg of intravenous fluids was administered but failed to improve hemodynamics. Continuous infusions of landiolol and norepinephrine were initiated thereafter. Sinus rhythm (SR) was restored approximately four hours after admission, coinciding with stabilization of HR and mBP.

**Figure 4:**
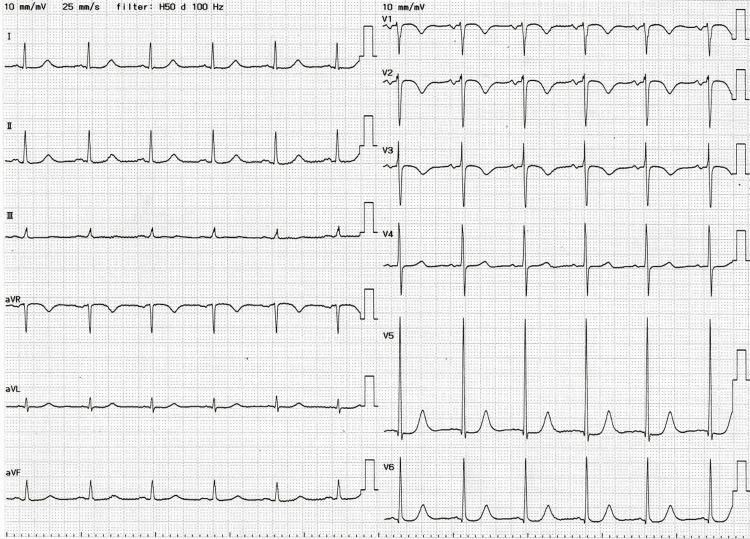
Electrocardiogram on hospital day two. The 12-lead ECG obtained on the day following admission demonstrates reversion to sinus rhythm. P waves are clearly visible preceding each QRS complex, and the ventricular rate is regular, confirming the restoration of atrioventricular conduction.

The patient's clinical timeline from presentation to the day following admission is illustrated in Figure 3. As his hemodynamics stabilized, landiolol and norepinephrine were gradually tapered and discontinued. On hospital day two, his blood pressure and heart rate remained stable, and a follow-up ECG confirmed reversion to sinus rhythm (Figure 4). Echocardiography performed on day two by the critical care team showed a preserved left ventricular ejection fraction (LVEF) of 50%, indicating no overt systolic dysfunction.

Despite cardiovascular stabilization, hypoxemia persisted, requiring ongoing oxygen therapy at 2 L/min. Continued management was carried out jointly by internal medicine and cardiology teams with a focus on treating CHF. Diuretic therapy was initiated, leading to the resolution of oxygen requirements by hospital day three.

**Figure 5:**
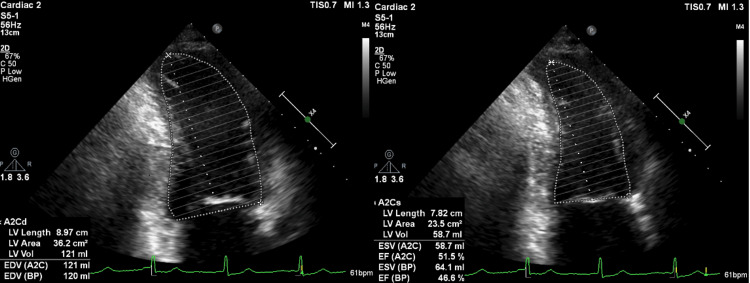
Follow-up transthoracic echocardiography on hospital day four. Transthoracic echocardiography in the apical two-chamber view was performed on hospital day four. The left image shows end-diastole and the right image shows end-systole. The calculated left ventricular ejection fraction (LVEF) was preserved at 46.6%, with no apparent valvular abnormalities or regional wall motion asynergy. Diffuse hypokinesis was noted, consistent with the clinical picture of transient cardiac dysfunction.

On day four, bisoprolol (0.625 mg), empagliflozin (10 mg), and spironolactone (25 mg) were introduced. Follow-up echocardiography on the same day revealed a reduced LVEF of 46.6% and diffuse left ventricular hypokinesis, without valvular abnormalities or regional wall motion asynergy (Figure 5). Coronary angiography performed on hospital day 13 revealed no significant coronary artery disease. The patient was discharged on hospital day 14 with continuation of the prescribed medications.

## Discussion

This is, to our knowledge, the first reported case of a cyanamide-ethanol reaction (CER) precipitating both new-onset atrial fibrillation (AF) and congestive heart failure (CHF) in a previously healthy individual.

Cyanamide and disulfiram are two primary agents used in alcohol deterrent therapy. Disulfiram irreversibly inhibits ALDH1 and ALDH2 through its metabolite diethyldithiocarbamate (DDC), with effects persisting for several days to a week. DDC also inhibits cytochrome P450 enzymes, increasing the risk of drug interactions and hepatotoxicity. Ethanol intake during disulfiram therapy can result in a disulfiram-ethanol reaction (DER) [6,7], occasionally leading to cardiogenic shock in overdose cases [8].

Cyanamide, by contrast, is metabolized in the liver to dicyanamide, which reversibly inhibits ALDH2. It has a more rapid onset (one to two hours) and a shorter duration of action (12-24 hours). Unlike disulfiram, it does not involve thiol radicals and thus presents a lower risk of hepatotoxicity and drug interactions.

Despite these pharmacologic advantages, the severity and variability of CER remain underrecognized. Fatal outcomes have been reported, particularly in Japan, and individual pharmacokinetic differences may prolong acetaldehyde accumulation beyond 12 hours after cyanamide intake, increasing the risk of severe reactions.

Acetaldehyde accumulation leads to peripheral vasodilation and sympathetic activation, which can provoke tachyarrhythmias [9]. Chronic alcohol use also contributes to atrial structural remodeling, predisposing patients to AF [10]. Moreover, acute alcohol intake has been implicated in the so-called “holiday heart syndrome,” characterized by new-onset AF in otherwise healthy individuals following moderate to heavy alcohol consumption. Although our patient ingested only a moderate amount of alcohol, it is plausible that a combination of autonomic surge, acetaldehyde toxicity, and underlying atrial vulnerability contributed to the sudden onset of AF, which in turn led to CHF.

Management of CER is primarily supportive, including fluid resuscitation and vasopressors to stabilize hemodynamics [3]. In cases of AF, rapid rate or rhythm control is essential. In our patient, early administration of a short-acting beta-blocker (landiolol) led to restoration of sinus rhythm and an improvement in blood pressure.

Long-term alcohol abstinence is associated with reduced recurrence of AF [11]. Therefore, multidisciplinary care involving internal medicine and psychiatry is essential to ensure both cardiovascular follow-up and sustained alcohol cessation. This case highlights the importance of recognizing CER as a cause of serious cardiovascular events, even when alcohol is consumed more than 12 hours after cyanamide administration.

## Conclusions

This case highlights the need to consider CER as a potential cause of acute atrial fibrillation and heart failure in patients undergoing cyanamide therapy, even when alcohol consumption occurs more than 12 hours after dosing and in modest amounts. CER can lead to serious cardiovascular complications and prolonged hospitalization. Given the possibility of persistent arrhythmias such as AF, clinicians should maintain a high index of suspicion and ensure appropriate long-term care, including interdisciplinary approaches to alcohol cessation support. Although the clinical course strongly suggests a causal relationship between CER and the development of atrial fibrillation and heart failure, we acknowledge that other potential triggers for arrhythmia could not be entirely ruled out.
